# Empiric Deworming and CD4 Count Recovery in HIV-Infected Ugandans Initiating Antiretroviral Therapy

**DOI:** 10.1371/journal.pntd.0003036

**Published:** 2014-08-07

**Authors:** Alexander J. Lankowski, Alexander C. Tsai, Michael Kanyesigye, Mwebesa Bwana, Jessica E. Haberer, Megan Wenger, Jeffrey N. Martin, David R. Bangsberg, Peter W. Hunt, Mark J. Siedner

**Affiliations:** 1 Department of Internal Medicine, Hospital of the University of Pennsylvania, Philadelphia, Pennsylvania, United States of America; 2 Department of Pediatrics, Children's Hospital of Philadelphia, Philadelphia, Pennsylvania, United States of America; 3 Center for Global Health, Massachusetts General Hospital, Boston, Massachusetts, United States of America; 4 Chester M. Pierce MD Division of Global Psychiatry, Department of Psychiatry, Massachusetts General Hospital, Boston, Massachusetts, United States of America; 5 Mbarara University of Science and Technology, Mbarara, Uganda; 6 University of California, San Francisco, San Francisco, California, United States of America; 7 Ragon Institute of Massachusetts General Hospital, Massachusetts Institute of Technology, and Harvard Medical School, Boston, Massachusetts, United States of America; 8 Division of Infectious Diseases, Department of Medicine, Massachusetts General Hospital, Boston, Massachusetts, United States of America; University of Washington, United States of America

## Abstract

**Background:**

There is conflicting evidence on the immunologic benefit of treating helminth co-infections (“deworming”) in HIV-infected individuals. Several studies have documented reduced viral load and increased CD4 count in antiretroviral therapy (ART) naïve individuals after deworming. However, there are a lack of data on the effect of deworming therapy on CD4 count recovery among HIV-infected persons taking ART.

**Methodology/Principal Findings:**

To estimate the association between empiric deworming therapy and CD4 count after ART initiation, we performed a retrospective observational study among HIV-infected adults on ART at a publicly operated HIV clinic in southwestern Uganda. Subjects were assigned as having received deworming if prescribed an anti-helminthic agent between 7 and 90 days before a CD4 test. To estimate the association between deworming and CD4 count, we fit multivariable regression models and analyzed predictors of CD4 count, using a time-by-interaction term with receipt or non-receipt of deworming. From 1998 to 2009, 5,379 subjects on ART attended 21,933 clinic visits at which a CD4 count was measured. Subjects received deworming prior to 668 (3%) visits. Overall, deworming was not associated with a significant difference in CD4 count in either the first year on ART (β = 42.8; 95% CI, −2.1 to 87.7) or after the first year of ART (β = −9.9; 95% CI, −24.1 to 4.4). However, in a sub-analysis by gender, during the first year of ART deworming was associated with a significantly greater rise in CD4 count (β = 63.0; 95% CI, 6.0 to 120.1) in females.

**Conclusions/Significance:**

Empiric deworming of HIV-infected individuals on ART conferred no significant generalized benefit on subsequent CD4 count recovery. A significant association was observed exclusively in females and during the initial year on ART. Our findings are consistent with recent studies that failed to demonstrate an immunologic advantage to empirically deworming ART-naïve individuals, but suggest that certain sub-populations may benefit.

## Author Summary

It is estimated that up to half of all people infected with HIV in sub-Saharan Africa are co-infected with one or more gastrointestinal parasites. These parasitic infections may negatively impact the ability of the immune system to combat the HIV virus, leading to worse clinical outcomes in people with HIV. Therefore, routine, universal, empiric treatment of gastrointestinal parasite infections (“deworming”) has been suggested as one strategy for optimizing HIV outcomes in this region. Previous studies have provided conflicting results on whether empiric deworming positively impacts markers of HIV disease progression such as CD4 count and viral load, but all of these studies were performed in HIV-infected individuals not yet on antiretroviral therapy. In this study, we measured the association between receipt of empiric deworming and CD4 count over time in HIV-infected adults taking antiretroviral therapy in southwestern Uganda, an area with a high parasite burden. We found that, overall, there was no significant association between deworming and change in CD4 count; however, when we performed a sub-analysis looking exclusively at females during the first year of ART, deworming was associated with significantly increased CD4 count. These results suggest that empiric deworming may not be an effective generalized strategy for improving HIV treatment outcomes in sub-Saharan Africa; however, the possibility of targeted benefit in specific sub-populations deserves further investigation.

## Introduction

Despite increased access to antiretroviral therapy (ART) in sub-Saharan Africa [1], HIV outcomes in this region have lagged behind those in more industrialized regions [2]. Although a complex interplay of economic, biologic, and sociobehavioral factors underlies this discrepancy, one potential contributing factor is the high rate of endemic, chronic, and overlapping parasitic co-infections, including schistosomiasis and the three major soil-transmitted helminth (STH) infections: *Ascaris lumbricoides*, *Trichuris trichiura*, and hookworms – caused by two species, *Ancylostoma duodenale* and *Necator americanus* [3]. A number of potential pathways have been proposed by which helminth infection might impair immunologic control of the HIV virus and accelerate disease progression. These include suppression of antiviral Th1 lymphocyte responses by helminth-driven Th2 lymphocyte skewing [4], [5], helminth-induced stimulation of other immunosuppressive cytokine responses [6], potentiation of cellular susceptibility to viral entry resulting from increased co-receptor expression [7], [8], and augmentation of systemic immune activation [3], [5], [9], [10]. A mechanism whereby helminth infection increases systemic immune activation is particularly intriguing, given the growing evidence that systemic inflammation and immune activation are associated with poor clinical outcomes in HIV-infected individuals on ART [10], [11], [12].

The geographic distributions of HIV and STH infection in sub-Saharan are largely overlapping [13]. Existing anti-helminthic drugs are well-tolerated, inexpensive, and easy to administer [14], [15]. On the other hand, barriers to diagnosing STH infection in resource-constrained settings are considerable [16], given that the current gold standard method of stool microscopy is both labor intensive and lacks optimal sensitivity [17], [18], [19], [20]. Furthermore, chronic gastrointestinal STH carriage is frequently asymptomic, or presents only with vague and non-specific manifestations that are common among HIV-infected persons – such as weight loss, nutritional deficiency, anemia, abdominal discomfort, or diarrhea [21], [22]. Therefore, routine empiric treatment of STH infection (“deworming”) in HIV-infected individuals living in regions with high prevalence of STH has been suggested as a potential intervention to delay HIV disease progression in ART naïve patients [4], [22], [23]. Studies testing this hypothesis in ART-naïve populations have been conflicting [24], [25], [26], [27]. A meta-analysis compiling data from three randomized controlled trials demonstrated a significantly lower plasma viral load and higher CD4 count in individuals receiving definitive therapy for confirmed helminth infection [28]. However, a more recent multi-site randomized controlled trial, reported by Walson and colleagues, demonstrated no beneficial impact of empiric deworming on markers of HIV disease progression in a similar ART-naïve population [29].

Most prior studies investigating the impact of empiric deworming on markers of HIV disease progression have been performed in ART-naïve individuals. There is a paucity of data to demonstrate the impact of deworming on clinical outcomes among HIV-infected persons taking ART. In the past decade over 8 million people living with HIV/AIDS have gained access to ART [1] and this number is expected to continue to grow rapidly as international guidelines recommend earlier treatment at higher CD4 counts [30]. There is increasing evidence that persistent immune activation – perhaps mediated to some extent by the translocation of pro-inflammatory microbial products across a dysfunctional gastrointestinal mucosal barrier [31], [32], [33] – is associated with mortality and adverse clinical outcomes, even among those achieving viral suppression on ART [11], [34], [35], [36], [37], [38]. In response, a number of interventions are in development and testing which aim to reduce immune activation in this population [39]. Given the potential associations between STH infection, disruption of the gut mucosal barrier, and immune activation, deworming therapy warrants further exploration as such an intervention.

Our primary objective was to estimate the association between empiric deworming and change in CD4 count over time in HIV-infected individuals receiving ART in southwestern Uganda. We hypothesized that receipt of deworming therapy (versus no antecedent deworming) was associated with greater increase in CD4 count. In the Mbarara district, where the majority of our study population resides, combined prevalence of the three major STH infections is estimated to be 20–50% [40], [41], [42], [43], [44]. Hookworm is the most commonly identified STH in epidemiologic surveys, both in this district and throughout East Africa [41], [42], [43]. Although schistosomal infection is endemic in many Ugandan communities living around low-lying bodies of fresh water, it is relatively uncommon in the higher altitude areas of Mbarara district [45], [46]. As a result, our clinic does not routinely perform diagnostic testing for *Schistosoma* species or administer empiric praziquantel.

A secondary objective was to estimate the association between empiric deworming and other markers of nutritional status, such as body mass and anemia, during treated HIV infection. Because STH infection is an important cause of malnutrition – specifically, hookworms are known to cause gastrointestinal blood loss resulting in iron deficiency anemia [47] – we hypothesized that deworming was associated with greater increase in total body mass and blood hemoglobin concentration with time on ART.

## Methods

### Ethics statement

Ethical approval for all study procedures was obtained from the Committee on Human Research, University of California at San Francisco; the Partners Human Research Committee, Massachusetts General Hospital; and the Institutional Review Committee, Mbarara University of Science and Technology. Consistent with national guidelines, we received clearance for the study from the Uganda National Council for Science and Technology and from the Research Secretariat in the Office of the President. The database used for this analysis is primarily a clinical database. Personal identifiers and protected health information are removed prior to data extraction and analysis. As such, all ethical review committees granted a waiver for informed consent.

### Study design and population

We conducted a retrospective, observational study among HIV-infected adults on ART at a large, publicly operated, regional HIV clinic located on the campus of the Mbarara Regional Referral Hospital in southwestern Uganda. Clinical data from 1998 through 2009 were analyzed in this study. Depending on clinical status, patients were seen in the clinic approximately two to six times annually. We included all subjects aged 17 years or greater who were on ART and had at least one recorded CD4 count test during the study period.

### Definition of variables

For a given CD4 count measurement, subjects were assigned as having received deworming treatment if they were prescribed either albendazole or mebendazole between 7 and 90 days before the date of the CD4 test. The same 7 to 90 day time window was used to assign credit for deworming with respect to the date of measurement for our secondary outcomes, body mass and hemoglobin. Standard practice in this clinic was to administer a single oral dose of either albendazole (500 milligrams) or mebendazole (400 milligrams) for routine deworming approximately once per year (which corresponds to approximately every six visits). We chose a 7 to 90 day window to assign credit for having received deworming therapy based on evidence that the likelihood of STH re-infection approaches 50% at 90 days post-treatment [48]. Additionally, this time window was chosen in order to maintain consistency with prior studies that examined the effect of deworming on CD4 count at 70 to 112 days after deworming [24], [25], [26], [27].

### Statistical analysis

We compared baseline characteristics between subjects who received at least one deworming treatment and those who received no deworming during the study period. Categorical variables were compared between these groups using chi squared testing, and continuous variables using the equality of medians test.

In our primary analysis, we fit a multivariable linear regression model to estimate the association between deworming and CD4 count. The primary explanatory variables of interest were time on ART, receipt (vs. non-receipt) of deworming, and an interaction between these two variables. Because graphical depiction of CD4 changes on ART demonstrated a clear leveling of the rate of increase in CD4 count after one year of ART, we implemented a linear spline model for duration of ART with a knot at one year. We then carried out a sub-analysis stratified by gender, to determine if there was a differential effect of deworming in males versus females. Finally, we performed two secondary analyses to estimate the association between deworming and 1) body mass (kilograms), and 2) blood hemoglobin concentration (grams per deciliter). We fit a separate multivariable linear regression model for each of these secondary outcomes, similarly using time on ART, receipt (vs. non-receipt) of deworming, and a time by deworming interaction term as our explanatory variables of interest.

To account for within-participant clustering over time, we used cluster-correlated robust estimates of variance [49], [50]. We adjusted for known correlates of HIV disease progression and CD4 count change, including age, time on ART, co-morbid tuberculosis infection, and pregnancy (if applicable). We considered also adjusting for known socio-demographic predictors of STH infection, such as educational attainment, socioeconomic status, and rural (versus urban) residence [18], [51]; however, in our clinical database these variables were inconsistently documented over the course of the study period. Given the high degree of missing data and the uneven distribution of missingness with respect to other subject characteristics, we did not include these additional co-variables in our final analyses. However, we did employ the following relevant variables in a sensitivity analysis: educational attainment, monthly income, and travel time from home to clinic.

To test the robustness of our primary model, we performed several sensitivity analyses. First, we fit a model in which CD4 count outliers were excluded. Outliers were defined as CD4 count measurements that varied by greater than 400 cells/mm^3^ (approximately +/−2 standard deviations from the median change in CD4 count) from the immediately previous or subsequent measurement. Additionally, we considered that the potential benefit of deworming on CD4 count may persist for greater than 90 days, and that some subjects who received deworming may have simply not been scheduled for a CD4 count measurement within the 7 to 90 day time window after deworming. Therefore, we performed analyses in which credit for deworming was extended to CD4 count measurements up 120 days and 180 days after treatment. Finally, although several compelling socio-demographic indicators were missing for the majority of our subjects, we performed a sensitivity analysis in which educational attainment, monthly income, and travel time from home to clinic were added as co-variables to our primary model in a step-wise fashion. For our secondary analyses, we performed sensitivity analyses in which we extended credit for deworming up to 120 and 180 days, stratified by gender, and excluded outliers, defined as body mass or hemoglobin measurements that were +/−3 standard deviations from the median. All data analysis was performed using Stata version 11.2 (StataCorp, College Station, Texas).

## Results

From 1998–2009, a total of 5,379 subjects on ART attended 21,933 clinic visits at which a CD4 count was measured. Sixty-one percent of subjects were female. The median baseline CD4 count and age at the time of ART initiation were 270 cells/mm^3^ and 38.3 years, respectively. During our study period, the median number of clinic visits per subject at which a CD4 count was measured was four. A total of 2,781 out of 5,379 (52%) subjects received deworming therapy at least once during the observation period, and a total of 668 (3%) CD4 count measurements were preceded by deworming therapy within the 7 to 90 day time window. Baseline characteristics, compared between subjects that received deworming therapy at least one time during the study period and those that received no deworming therapy during the study period, are summarized in Table 1.

**Table 1:** Subject characteristics.

Characteristic	n	Received deworming at least once during study period (n = 2781)	Never received deworming during study period (n = 2598)	χ2 (p-value)
Gender; n (%)	5379			
Female	3302	1695 (61.0%)	1607 (61.9%)	0.47 (0.50)
Male	2077	1086 (39.0%)	991 (38.1%)	
Time of ART initiation; n (%)	5379			
Prior to January 1, 2007	2868	1482 (53.3%)	1386 (53.4%)	0.002 (0.97)
On or after January 1, 2007	2511	1299 (46.7%)	1212 (46.6%)	
Baseline CD4 count at time of ART initiation; median (IQR)	5379	265 (163–392)	273 (166–395)	1.72 (0.19)
Age at time of first post-ART CD4 count (years); median (IQR)	5359	38.4 (32.5–44.3)	38.0 (32.4–44.7)	1.42 (0.23)
Clinic visits at which CD4 count was obtained; median (IQR)	5379	4 (2–6)	3 (2–5)	90.49 (<0.001)
Education; n (%)	2106			
Primary only	1457	847 (69.9%)	610 (68.2%)	0.77 (0.38)
Secondary or greater	649	364 (30.1%)	285 (31.8%)	
Monthly income (Uganda shillings)*; n (%)	1439			
<100000	1095	616 (76.1%)	479 (76.0%)	0.002 (0.96)
≥100000	344	193 (23.9%)	151 (24.0%)	
Self-reported travel time from home to clinic	1577			
<1 hour	815	464 (52.6%)	351 (50.5%)	0.69 (0.41)
>1 hour	762	418 (47.4%)	344 (49.5%)	
Diagnosed with TB at least once during study period	5379			
Yes	1033	540 (19.4%)	493 (90.0%)	0.17 (0.68)
No	4346	2241 (80.6%)	2105 (81.0%)	
Pregnant at least once during study period	3302			
Yes	733	375 (22.1%)	358 (22.3%)	0.01 (0.92)
No	2569	1320 (77.9%)	1249 (77.7%)	

*100000 Uganda shillings valued at approximately 40 USD as of April 1, 2014.

**Figure 1:**
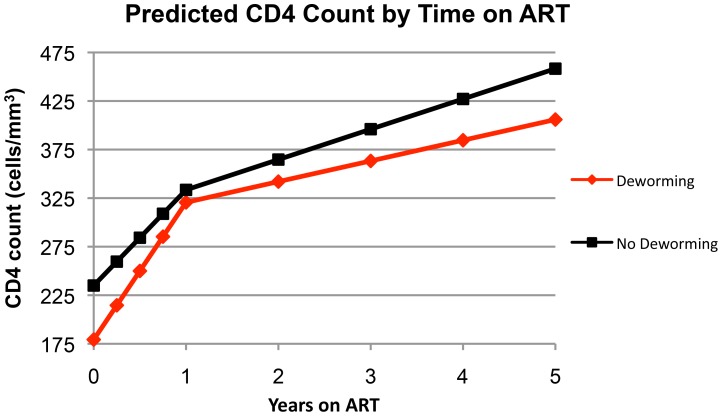
Predicted CD4 count by time on ART. Predicted values are based on the multivariable linear regression model **y = α+β_1_t+β_2_t+β_3_t+β_4_t**, where the independent variable, *y*, is CD4 count; the dependent variable, *t*, is time on ART, the *y*-intercept, α, is CD4 count at *t* = 0, and **β_1_**–**β_4_** are respective **β**-coefficients for the co-variables age, TB co-infection, deworming, and deworming*time interaction.

In our primary multivariable linear regression analysis (Table 2), deworming was not significantly associated with a change in CD4 count over time in either the first year on ART (β = 42.8; 95% CI, −2.1 to 87.7) or after the first year of ART (β = −9.9; 95% CI, −24.1 to 4.4). In this model, statistically significant predictors of CD4 count were age, time on ART, tuberculosis co-infection, and receipt of deworming. Based on estimates derived from our primary model, predicted CD4 count as a function of time on ART is graphically depicted in Figure 1. In our sensitivity analyses, excluding outlier CD4 values, extending the treatment window, or adjusting for additional socio-demographic variables did not yield qualitatively different results.

**Table 2:** Primary analysis: multivariable linear regression model of predictors of CD4 count (n = 5379).

Parameter	β	95% CI	p-value
Time on ART			
0 to 1 year (per year of ART up to 1 year)	98.5	85.5 to 111.6	<0.001
>1 year (per year of ART after 1 year)	31.2	26.8 to 35.6	<0.001
Age (each year of age)	−0.8	−1.4 to −0.2	0.011
TB co-infection	−114.8	−153.9 to −75.8	<0.001
Deworming	−55.6	−86.3 to −25.0	<0.001
Deworming×Time on ART interaction term†			
0 to 1 year on ART	42.8	−2.2 to 87.7	0.062
>1 year on ART	−9.9	−24.1 to 4.4	0.174

† Predicted difference in CD4 count between patients receiving versus not receiving deworming therapy in the past 90 days. The interaction terms were separated by duration of prior ART use as up to 1 year of therapy versus greater than 1 year of therapy.

In the sub-analysis restricted to female gender (Table 3), deworming was significantly associated with a greater increase in CD4 count during the first year of ART (β = 63.0; 95% CI, 6.0 to 120.1), but not after the first year of ART (β = −15.4; 95% CI, −32.6 to 1.8). Statistically significant predictors of CD4 count were time on ART, tuberculosis co-infection, pregnancy, and receipt of deworming. When the analysis was restricted to male gender, deworming was not significantly associated with CD4 count change in either the first year on ART (β = 13.0; 95% CI, −57.4 to 83.4) or after the first year of ART (β = −6.9; 95% CI, −29.3 to 15.5). Statistically significant predictors of CD4 count for males were time on ART and tuberculosis co-infection. Notably, as compared to males in our study population, females were significantly younger (median age 36.2 vs. 41.3; p<0.001), more likely to have initiated ART after 2007 (48.1% vs. 44.4%; p = 0.009), less likely to have ever had tuberculosis during the study period (15.2% vs. 25.6%; p<0.001), and had significantly higher pre-ART baseline CD4 count (282 vs. 248; p<0.001). Females and males were not significantly different with respect to the likelihood of receiving deworming at least once during the study period, as well as the median number post-ART visits at which a CD4 measurement was obtained.

**Table 3:** Multivariable linear regression model of predictors of CD4 count, stratified by gender (n = 5379).

	Female (n = 3302)			Male (n = 2077)		
Parameter	β	95% CI	p-value	β	95% CI	p-value
Time on ART						
0 to 1 year (per year of ART up to 1 year)	109.8	92.5 to 127.0	<0.001	65.6	45.2 to 86.0	<0.001
>1 year (per year of ART after 1 year)	38.4	32.1 to 44.7	<0.001	22.6	16.9 to 28.4	<0.001
Age	0.1	−0.8 to 1.0	0.777	0.3	−0.6 to 1.1	0.578
TB co-infection	−104.4	−161.3 to −47.4	<0.001	−109.5	−159.0 to −60.1	<0.001
Pregnant	−31.8	−53.1 to −10.7	0.003	n/a	n/a	n/a
Deworming	−67.3	−103.9 to −30.7	<0.001	−34.3	−88.7 to 20.1	0.217
Deworming×Time on ART interaction†						
Deworming during 0 to 1 year on ART	63.0	6.0 to 120.1	0.030	13.0	−57.4 to 83.4	0.717
Deworming during >1 year on ART	−15.4	−32.6 to 1.8	0.078	−6.9	−29.3 to 15.5	0.544

† Predicted difference in CD4 count between patients receiving versus not receiving deworming therapy in the past 90 days. The interaction terms were separated by duration of prior ART use as up to 1 year of therapy versus greater than 1 year of therapy.

In the secondary analyses, there was no significant effect of deworming on either body mass (β = −0.09; 95% CI, −0.40 to 0.23) or hemoglobin (β = 0.08; 95% CI, −0.05 to 0.22) over time (Table 4). Statistically significant predictors of both body mass and hemoglobin were time on ART, age, tuberculosis co-infection, and receipt of deworming. The sensitivity analysis in which we extended the time window for assigning deworming credit and simultaneously restricted the population to females resulted in the new observation that deworming was significantly associated with increased hemoglobin concentration over time for both the 120 day (β = 0.15; 95% CI, 0.02 to 0.27) and the 180 day (β = 0.14; 95% CI, 0.04 to 0.25) time windows. Otherwise, the remaining sensitivity analyses for both the body mass and hemoglobin outcomes yielded qualitatively similar results to the original models.

**Table 4:** Secondary analysis: multivariable linear regression modeling of predictors of body mass and blood hemoglobin concentration.

Parameter	β	95% CI	p-value
**Body Mass (kg), n = 7225**			
Time on ART (years)	1.11	0.97 to 1.25	<0.001
Age (each year of age)	0.08	0.05 to 0.11	<0.001
TB co-infection	−4.95	−5.88 to −4.03	<0.001
Deworming	−2.43	−2.99 to −1.87	<0.001
Deworming×Time on ART interaction term†	−0.09	−0.40 to 0.23	0.599
**Hemoglobin (g/dl), n = 4811**			
Time on ART (years)	0.33	0.30 to 0.37	<0.001
Age (each year of age)	0.01	0.00 to 0.02	0.001
TB co-infection	−1.67	−2.37 to −0.97	<0.001
Deworming	−0.81	−1.15 to −0.47	<0.001
Deworming×Time on ART interaction term†	0.08	−0.05 to 0.22	0.222

† Predicted difference in body mass or blood hemoglobin concentration between patients receiving versus not receiving deworming therapy in the past 90 days.

## Discussion

In this retrospective analysis accounting for over 600 deworming events in more than 5,000 persons accessing HIV care at a public clinic during a 10 year period in rural Uganda, we found no generalized benefit of empiric deworming on CD4 count recovery among HIV-infected individuals on ART. Although it did not reach statistical significance, it is notable that deworming predicted an increased CD4 count over time during the first year on ART, but not during subsequent years. Additionally, in the sub-population of females, deworming predicted a significantly greater rise in CD4 count during the first year on ART. Not only was the latter effect statistically significant, but compared to our primary model the magnitude of CD4 count increase was greater. In the secondary analysis, deworming conferred no benefit on body weight over time. Similarly, in our original model for the hemoglobin outcome, deworming conferred no benefit on hemoglobin level over time; however, we did observe a small but significant increase in hemoglobin level with deworming when we concurrently restricted the population to females and extended the time window for assigning deworming credit to 120 or 180 days before a given hemoglobin measurement.

Interestingly, in both our primary model and the sub-analysis restricted to females, baseline CD4 count at time of ART initiation was significantly lower in subjects who received deworming. One possible explanation for this would be if clinicians were more likely to recommend deworming to patients who were judged to be clinically more ill, or more specifically to those with lower CD4 counts. This notion is supported by our finding that both baseline body weight and hemoglobin were also lower in subjects who received deworming. Given that we observed a non-significant trend toward greater CD4 count recovery during the first year of ART with deworming (and a significant effect in the female sub-analysis), it is intriguing to speculate that our estimate did not reach significance simply due to methodological limitations, such as an unidentified bias toward the null, under-powering, or residual confounding. Additionally, the dominant effect of ART on CD4 count recovery may have masked any smaller, but still potentially significant, effect of deworming. In light of these limitations, it is critical to interpret the above findings in the context of the disparity in baseline CD4 count between those receiving deworming and those not (Table 1). The fact that, independent of deworming, there was no significant association between baseline CD4 count and rate of CD4 count rise during the first year of ART (data not shown) suggests that a true treatment effect may be more likely.

It should be noted that females in our study population differed significantly from males with respect to several important baseline characteristics in our model, including age, tuberculosis co-infection, and baseline CD4 count. Our finding that deworming had a significant benefit for females during the first year on ART may be partly explained by the fact that females in our population were younger and less likely to have tuberculosis co-infection, since both age and tuberculosis co-infection were inversely associated with CD4 count in our primary model, and in prior studies [52], [53]. Notably, our models included both age and tuberculosis co-infection, which should account for potential confounding by these factors.

We are unaware of prior data supporting a differential impact of deworming between males and females. The most likely explanation is unmeasured confounding between these two groups. Barring that, a plausible explanation for our findings would be greater prevalence of helminth infection or higher parasite load among females in our population. However, we have no compelling reason to suspect this. In fact, several studies in HIV-infected populations have demonstrated that prevalence of STH infection in some areas of East Africa is actually similar to or lower in females than in males [54], [55], [56], [57]. However, that these studies were conducted in regions outside of our geographic area of scope and may not be generalizable to our study population.

Models demonstrating the impact of deworming on changes in body mass and hemoglobin after ART initiation paralleled the results of our primary analysis of CD4 count. Although both models failed to demonstrate a significant effect of deworming, time on ART was strongly associated with both greater body mass and greater hemoglobin levels. Similarly, tuberculosis co-infection was strongly associated with both decreased body mass and decreased hemoglobin level. Taken together, these findings support the validity of the analyses because they are consistent with anticipated results based on known relationships.

Interestingly, in a sensitivity analysis in which we permitted an increased duration of deworming therapeutic response from 90 to 120 or 180 days, we found that deworming was associated with increases in hemoglobin in females only. This finding may be explained by the fact that hemoglobin levels are checked less frequently than CD4 count in our clinical population. Therefore, lengthening the deworming therapeutic time window may have increased the number of hemoglobin measurements receiving credit for deworming, thereby increasing the statistical power to allow for measurement of this association. Alternatively, it is possible that a period of time greater than 90 days is required for deworming to sufficiently exert an effect on hemoglobin levels, which is consistent with the known red blood cell life span of approximately 120 days. Alternatively, deworming may confer a benefit on hemoglobin levels directly by reducing the gastrointestinal parasite load, particularly with respect to hookworms.

Although this study is strengthened by its large sample size (668 deworming visits) and ability to adjust for key confounding variables, we acknowledge that there are several notable limitations beyond the inherent risks for confounding and bias in a retrospective analysis. First, although STH infection is reported to be high in southwestern Uganda [40], [41], [42], [43], [44], actual prevalence in our study population was unknown. Lower rates of infection than previously published would bias the observed effect of any true deworming treatment effect towards the null. Similarly, STH infection intensity (stool parasite load) was also unmeasured, although it should be considered as a factor that may modulate any potential effect of empiric deworming. The intensity of STH infection has been shown to correlate with certain markers of clinical disease severity, including anemia and eosinophilia [18], [19]. Therefore, it is conceivable that deworming may exert a greater beneficial effect on CD4 count recovery in the setting of higher intensity STH infection. Second, because viral load monitoring was not routinely available in our study setting, it is not possible to adjust for the presence or absence of viral suppression, an important predictor of CD4 reconstitution. We would only expect confounding if receipt of deworming therapy were associated with virologic failure, which is unlikely given the nearly equivalent patterns of CD4 reconstitution observed in both groups. Third, a small incremental effect conferred by deworming may be obscured by the expected dominant effect of ART (and resulting suppression of HIV activity) on CD4 count recovery. Fourth, although most patients (52%) received deworming therapy at least once during the observation period, deworming therapy was recorded prior to only 3% of visits with a corresponding CD4 count measurement, which is less than what would be expected with annual deworming visits (i.e. closer to 10–20%). Fifth, patients at our clinic site receive only a single dose of either mebendazole or albendazole at each deworming. Therefore, it is possible that high rates of STH re-infection [48] and incomplete eradication of parasite load with standard doses of anti-helminthic therapy [58], especially in individuals with a large parasite burden, may obscure the potential benefit of deworming on CD4 count recovery.

Finally, host-parasite interactions during STH infection are complex and incompletely understood. In addition to causing disease, helminths have been associated with protection from atopic syndromes and other pathologic inflammatory conditions [59], [60]. Like these conditions, untreated HIV infection is characterized by the presence of exuberant systemic inflammation and immune activation. This lessens with the initiation of ART, but never completely normalizes to the levels seen in HIV-uninfected individuals [12], [36]. Furthermore, even in the setting of virologic control on ART, higher levels of immune activation are associated with increased risk for HIV disease progression and mortality [11], [12], [33], [36]. Given that there are conflicting data on the association between STH infection and immune activation [3], [5], [61], [62], it is plausible to consider that STH infection may promote immune activation only in certain circumstances. This is supported by evidence that STH infections may both promote and protect against immune activation – by disrupting the gut mucosal barrier during high-intensity infection, or by reinforcing mucosal barriers in other settings, respectively [63].

Therefore, we cannot rule out the possibility that, depending on host and parasite-specific characteristics (for example: helminth species, stage of infection, host genetic factors), STH infection – and therefore deworming – may alternately confer either beneficial or deleterious effects on HIV disease progression. Indeed, it has been suggested that low-intensity infection with certain helminth species may protect against HIV disease progression [5], [64]. Thus, it is possible that only under certain circumstances will deworming confer a significant benefit on HIV disease progression or CD4 count recovery. Under certain conditions, deworming could even cause harm by disrupting as yet incompletely understood pathways by which helminths protect against pathologic inflammation and T-cell activation. Alternatively, anti-helminthic agents could have pleiotropic effects with either antiviral or immunomodulatory properties.

One might consider a hypothetical model in which deworming is beneficial during the most active period of STH-mediated mucosal disruption (such as early in the natural history of STH infection), but is in fact detrimental or has no effect during chronic, stable, low-intensity infection – when protective anti-inflammatory or mucosal barrier stabilizing effects of STH infection presumably predominate. Consistent with this model, the variation in life cycle and time course of infection between different STH species may explain species-specific differences in the effect of deworming on HIV disease progression that have been observed in certain settings [24], [55]. Furthermore, several studies have demonstrated an association between STH infection and higher CD4 count in ART-naïve, HIV-infected individuals [18], [51], [54]. Although potentially confounded by a number of factors, this finding is consistent with a model in which low-intensity STH infection confers a beneficial effect on CD4 count.

In summary, our findings are consistent with previous studies that failed to find a benefit of empiric deworming in ART-naïve, HIV-infected individuals [25], [29], [55], but also suggest that there may be a modest benefit among women on ART. Although our study does not support a role for universal, empiric deworming as a method to improve immune reconstitution in the general population of HIV-infected persons on ART, this strategy warrants further investigation as a potential adjunct to optimize ART effectiveness in females. Regardless of these results, deworming should continue to be a fundamental part of routine care for individuals living in areas highly endemic for STH infection.

Given the inherent constraints of our retrospective study design, including the inability to select at least a representative sample of subjects with confirmed and quantified STH infection, there is a need for more thoroughly controlled, randomized, and prospective studies that will avoid such limitations. These future investigations could more clearly establish any deworming treatment effects of targeted benefit to those with confirmed STH infections. Future studies should also examine the role of deworming using optimally effective anti-helminthic drug regimens, the effect of empiric treatment on specific sub-populations, and the impact of deworming on other surrogate and clinical markers or sequelae of HIV disease, including immune activation, systemic inflammation, gut microbial translocation, and cardiovascular disease. Additional investigations are also warranted to confirm our finding of improved CD4 count recovery with empiric deworming in the sub-population of females.

Finally, to our knowledge this is the first study to describe the impact of deworming on clinical and immunologic markers of HIV disease in patients on ART. Given the increasing accessibility of ART in sub-Saharan Africa and other regions that are co-endemic for HIV and STH infection, future research on deworming and HIV infection ought to focus on patients initiating or already on ART.

## Supporting Information

Checklist S1 STROBE Checklist. Check (√) marks indicate that the authors have included the item in the manuscript. “N/A” indicates that the authors feel the item is not applicable to this specific study.
(PDF)
